# Comparison of Inflammatory Response to Transgastric and Transcolonic NOTES

**DOI:** 10.1155/2016/7320275

**Published:** 2016-06-14

**Authors:** Tomas Hucl, Marek Benes, Matej Kocik, Alla Splichalova, Jana Maluskova, Martin Krak, Vera Lanska, Marie Heczkova, Eva Kieslichova, Martin Oliverius, Julius Spicak

**Affiliations:** ^1^Department of Gastroenterology and Hepatology, Institute for Clinical and Experimental Medicine, Videnska 9, 14021 Prague, Czech Republic; ^2^Department of Transplantation Surgery, Institute for Clinical and Experimental Medicine, Videnska 9, 14021 Prague, Czech Republic; ^3^Institute of Microbiology, Academy of Science, Videnska 1083, 14021 Prague, Czech Republic; ^4^Department of Pathology, Institute for Clinical and Experimental Medicine, Videnska 9, 14021 Prague, Czech Republic; ^5^Department of Anesthesiology, Institute for Clinical and Experimental Medicine, Videnska 9, 14021 Prague, Czech Republic; ^6^Department of Statistics, Institute for Clinical and Experimental Medicine, Videnska 9, 14021 Prague, Czech Republic

## Abstract

*Aims.* The aim of our study was to determine the physiologic impact of NOTES and to compare the transgastric and transcolonic approaches.* Methods.* Thirty pigs were randomized to transgastric, transcolonic, or laparoscopic peritoneoscopy. Blood was drawn and analyzed for C-reactive protein (CRP), tumor necrosis factor-*α* (TNF-*α*), interleukin- (IL-) 1*β*, IL-6, WBCs, and platelets.* Results.* Endoscopic closure with an OTSC was successful in all 20 animals. The postoperative course was uneventful in all animals. CRP values rose on day 1 in all animals and slowly declined to baseline levels on day 14 with no differences between the groups (*P* > 0.05, NS). The levels of TNF-*α* were significantly increased in the transcolonic group (*P* < 0.01); however this difference was already present prior to the procedure and remained unchanged. No differences were observed in IL1-*β* and IL-6 values. There was a temporary rise of WBC on day 1 and of platelets on day 7 in all groups (*P* > 0.05, NS).* Conclusions.* Transgastric, transcolonic, and laparoscopic peritoneoscopy resulted in similar changes in systemic inflammatory markers. Our findings do not support the assumption that NOTES is less invasive than laparoscopy.

## 1. Introduction

Minimally invasive surgery has been challenging traditional open surgery. Laparoscopic surgery is less traumatic than open surgery and is generally associated with fewer local and systemic complications, less postoperative pain, and faster recovery [1]. Natural Orifice Translumenal Endoscopic Surgery (NOTES) is a novel technique that takes advantages of natural orifices [2]. The advantages of NOTES have not yet been fully confirmed in randomized trials but are expected to include, apart from improved cosmesis and absence of hernia, a less profound systemic response [3]. To date, a variety of surgical procedures using natural orifices have been performed in animals and humans. The postoperative course has not differed much from that of laparoscopic surgery, but these findings remain to be confirmed in controlled trials [3–7]. For example, the German NOTES registry, the largest registry worldwide, currently lists over 4100 procedures. In a recent analysis from this registry, the results of 217 transgastric and transvaginal NOTES appendectomies were reported, showing a short operating time, low complication rate, and a reasonable conversion rate [8].

Furthermore, NOTES has resulted in the development of new endoscopic and surgical procedures such as peroral endoscopic myotomy (POEM), peroral pyloromyotomy (POP), endoscopic full-thickness resection (EFTR), submucosal tunnel endoscopic resection (STEM), or transanal minimally invasive surgery for total mesorectal excision (TAMIS-TME). For some of these procedures, positive outcome data are rapidly accumulating [9].

In NOTES procedures, an endoscope enters the abdominal cavity by way of various orifices including the stomach, colon, or vagina. The choice of orifice is primarily determined by the type and location of procedure; however there is a general fear of infectious complications if the colon is used. The vaginal route with manual suture closure has been used in most human studies but is limited to only half of the population and has raised concerns of possible functional consequences [3, 4, 6, 10].

Ultimately, the adoption of NOTES in clinical practice depends on demonstrating its benefits including decreased impact on physiological and immunological status. The few studies that have evaluated the impact of NOTES on inflammatory response reported variable and occasionally conflicting results. For example, a late decrease in serum tumor necrosis factor-*α* (TNF-*α*) in pigs following transgastric peritoneoscopy was reported by McGee et al. [11]. However this was not confirmed in peritoneal fluid in a subsequent study by the same authors [12] and was contradicted in a study by Bingener et al., who reported a late TNF-*α* elevation in pigs undergoing NOTES [13].

The aim of our study was to investigate the impact of NOTES on the inflammatory response to surgical trauma caused by the procedure and to compare the impact with use of different sites of transluminal access.

## 2. Methods

### 2.1. Animals and Preparations

All animal experiments were approved by the Animal Care and Use Committee of the Institute of Clinical and Experimental Medicine and conducted in compliance with local and European guidelines.

The study was performed in female pigs weighing between 23 and 40 kg. The animals were randomized using computer generated random numbers to peritoneoscopy by transgastric NOTES, transcolonic NOTES, or laparoscopy. Animals were on a liquid diet and fasted overnight before surgery, and those in the transcolonic group were given a polyethylene glycol preparation the day before the procedure. Randomization was done prior to the bowel prep; thus only animals in the transcolonic group underwent bowel cleansing. Prior to endoscopy, ketamine (10 mg/kg) and azaperone (4 mg/kg) were injected intramuscularly; animals were endotracheally intubated, and general anesthesia was induced and maintained with isoflurane (0.8–1.5%) and fentanyl (3–5 mL/h). One dose of cefazolin (1 g) was administered intravenously 30 minutes before endoscopy.

### 2.2. Procedures

All procedures were performed under aseptic conditions. For transluminal procedures, a standard double-channel gastroscope (CF 2T160I; Olympus Optical Co., Tokyo, Japan) was used. The stomach/colon was irrigated with 1 L of disinfectant solution (10% Betadine; Egis Pharmaceuticals, Budapest, Hungary) and the site of access on the anterior wall of the stomach/colon was determined with the help of abdominal wall transillumination. A Veress needle was introduced percutaneously into the peritoneal cavity in the umbilical region using standard techniques; pneumoperitoneum was established and continuously maintained using a carbon dioxide insufflator with a built-in manometer set at 15 mm Hg (UHI-3, Olympus).

A gastric wall incision was made with a triple lumen needle knife (Microknife XL, Boston Scientific, Natick, Massachusetts, USA) followed by balloon dilation with 18 mm controlled radial expansion (CRE) balloon (Boston Scientific). The endoscope was advanced to the peritoneal cavity, which was thoroughly explored for 15 minutes. Visualization of the liver, stomach, spleen, small and large bowel, and urinary bladder was attempted.

Gastric/colonic access site closure was done with an over-the-scope clip (OTSC, Ovesco, Germany) and a double grasper (Ovesco) using the manufacturer-recommended techniques. Briefly, after mounting the delivery system onto the endoscope, one arm of the grasper anchored one edge of the incision and the second arm anchored the edge on the other side. Pulling the grasper while desufflating air brought both grasped edges of the incision into the transparent cap of the delivery system. The clip was then released. Patency of the incision was evaluated by intra-abdominal pressure monitoring after air insufflation in the stomach for at least 1 min.

Laparoscopic surgery was performed using standard techniques with one 10 mm port for a camera placed at the umbilicus and one 5 mm port for a grasper placed at the midline approximately 5 cm above the umbilicus. A 15 min peritoneoscopy was performed with CO_2_ at a pressure of 15 mm Hg (the same as for NOTES animals) produced by an automated insufflator.

### 2.3. Postoperative Period

After recovery, pigs were transferred to an animal facility where they were evaluated daily for complications. They were fed a standard swine feeding mixture starting on the first postoperative day. For postoperative analgesia, the animals were given fentanyl (50 *μ*g/h) by means of a dermal patch for 3 days. After a follow-up period of 14 days, they were sacrificed using identical methods of anesthesia followed by administration of KCl (30–50 mL). After endoscopic evaluation of the closure site, thorough exploration of the peritoneal cavity for signs of complications was performed. Tissue samples were taken from the closure site for histological examination.

### 2.4. Laboratory Investigations

Blood was drawn immediately before and 2 h, 1, 2, 7, and 14 days after the procedure. Whole blood was used for blood cell counts; the samples were analyzed immediately using an automated analyzer. For CRP and cytokine analysis, the blood was centrifuged; the serum was immediately frozen at −80°C and stored for later processing.

Serum levels of CRP, interleukin- (IL-) 1*β*, IL-6, and TNF-*α* were determined by ELISA with sensitivities of 10 ng/mL, 11 pg/mL, 12 pg/mL, and 13 pg/mL, respectively. Assays were performed with a commercially available CRP kit (Tridelta Development, Maynooth, Ireland) and matched-pair antibody sets for IL-1*β*, IL-6 (R&D Systems, Minneapolis, MN, USA), and TNF-*α* (Life Technologies, Carlsbad, CA, USA) at the end of the collection period. Whenever required, a pig-specific assay was used. Each test for every time-point and animal was done in triplicate at appropriate dilutions and with corresponding controls.

### 2.5. Statistical Analysis

For continuous variables, between- and within-group differences were compared using a general mixed-model analysis of variance (ANOVA). Group means were compared by the *t*-test for continuous variables and the Fisher exact test for discrete variables with the Bonferroni correction when appropriate. Highly skewed variables were log-transformed before analysis. A two-tailed *P* < 0.05 was required for statistical significance. Analyses were performed using JMP statistical software (SAS Institute, USA).

## 3. Results

### 3.1. Procedure and Closure

Access of the peritoneal cavity was gained in all NOTES and laparoscopy animals without complications. All the targeted organs in the peritoneal cavity were visualized in all animals, and peritoneoscopy revealed no signs of access-related damage to adjacent organs.

**Figure 1:**
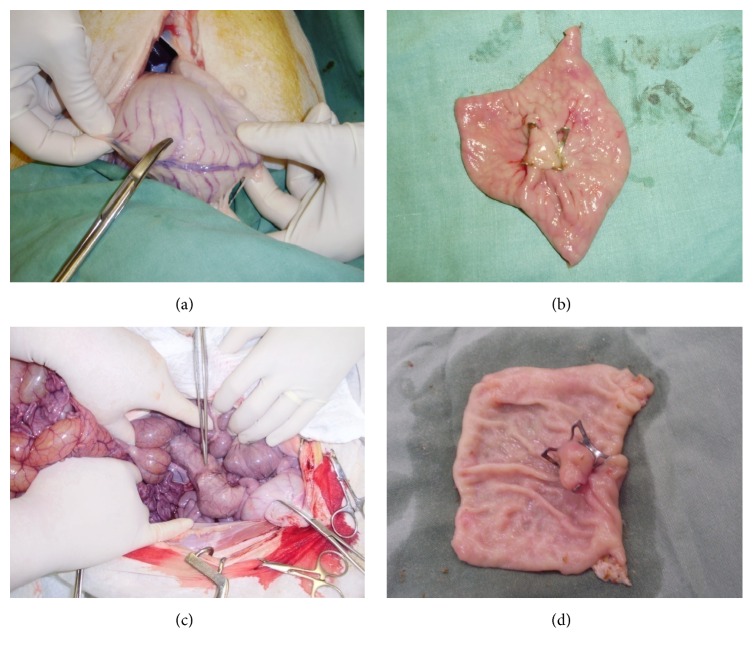
OTSC closure in stomach and colon at autopsy. (a) Serosal site of stomach closure. (b) Mucosal site of stomach closure. (c) Serosal site of colon closure. (d) Mucosal site of colon closure.

Gastric incision-site closure was successfully performed with an OTSC in all 10 animals in a mean time of 8 min (range 4–12 min; Figure 1, Table 1). Colonic incision-site closure was successfully performed with an OTSC in all 10 animals in a mean time of 6 min (range 4–9 minutes; Figure 1, Table 1). Endoscopic appearance of a complete closure was always achieved. At the end of each procedure, we observed full distention of the stomach/colon on air insufflation in all animals with no rise in the intra-abdominal pressure.

Peritoneal access using two trocars, laparoscopic peritoneoscopy, and hand suturing of incisions proceeded uneventfully. All the targeted organs were visualized.

### 3.2. Postoperative Period

The animals recovered well and resumed their regular diet on the first postoperative day. During the 2-week follow-up period, we observed no clinical signs suggestive of complications. All animals gained weight with no differences between the groups (data not shown).

### 3.3. Necropsy and Histological Examination

Endoscopic examination before necropsy revealed normally appearing stomach/colon, which was easily fully distended upon insufflation in all animals, and the site of closure appeared to be well healed without signs of gross ulceration or inflammation (Figure 1). The clips were still in place in 8 of 10 animals in the transgastric group and in all animals in the transcolonic group. At necropsy, there were no signs of organ damage or inflammation in the peritoneal cavity, and the serosal side of incision closure appeared to be well healed. Minor adhesions were observed at the gastric site closure in 4 animals and at the colonic site closure in 2 animals, whereas major adhesions between bowel loops were seen in 2 animals in the transcolonic group (*P* = 0.24, Table 1).

**Figure 2:**
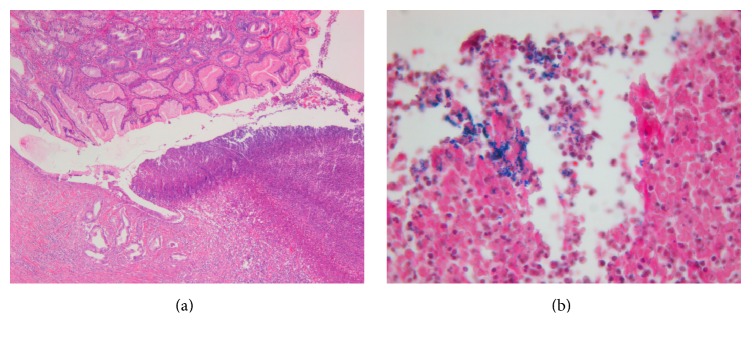
Histopathology of closure site (H&E, 50x). (a) Purulent exudate at site of closure, 10x. (b) Gram-positive staining at closure site, 20x.

Histopathological examination of the excised closure sites revealed transmural healing in all animals. However, 4 animals in the transgastric group and 3 animals in the colonic group had signs of purulent exudates in the mucosa with positive Gram staining (*P* = 1, Figure 2).

### 3.4. TNF-*α*

**Figure 3:**
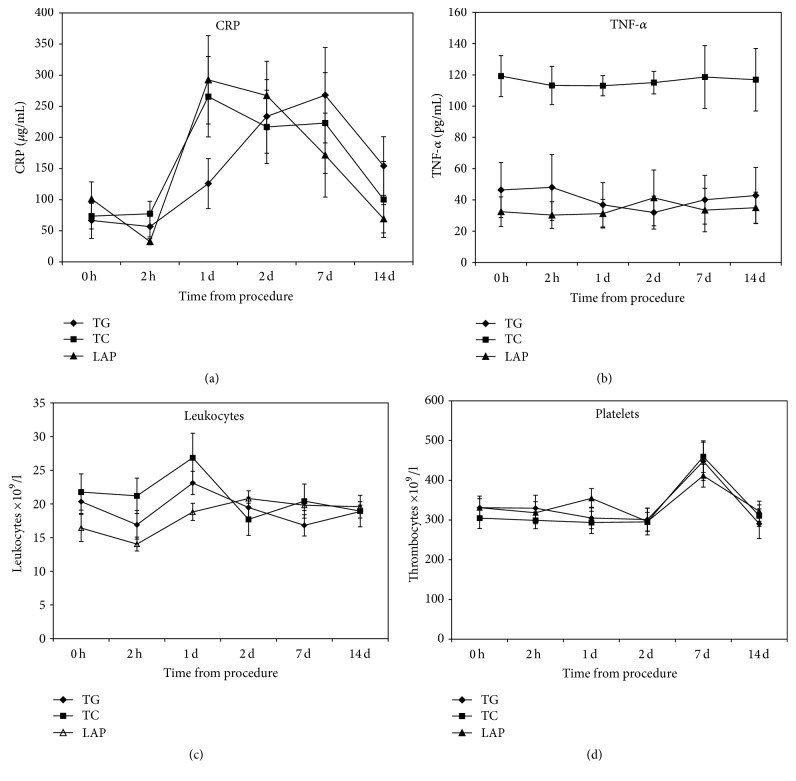
Comparison between transgastric, transcolonic, and laparoscopic peritoneoscopy in levels of (a) C-reactive protein; (b) TNF-*α*; (c) leukocytes; (d) platelets. Values are shown as means and vertical bars representing SEM.

The TNF-*α* remained practically unchanged for the entire period of observation in all animals. It was significantly higher in the colonic group than in the transgastric and laparoscopic groups (*P* < 0.01); however this difference was already present prior to the procedure and remained unchanged. No other differences were seen (Figure 3).

### 3.5. IL-1*β* and IL-6

IL-1*β* levels were detectable in a minority of animals (transgastric, 12%; transcolonic, 2%; laparoscopic 8%). IL-6 levels were detectable only in some animals (transgastric, 63%; transcolonic, 43%; laparoscopic, 23%). In those with a measurable result, the levels remained constant over time and were not statistically different between the groups (data not shown).

### 3.6. C-Reactive Protein

CRP values rose on day 1 in all animals. This elevation persisted until the seventh postoperative day and then slowly decreased toward baseline levels on day 14 with no statistically significant differences between the groups (Figure 3).

### 3.7. Leukocytes and Platelets

There was a rise in leukocyte count in all animals that peaked on day 1 in the NOTES animals and on day 2 in laparoscopy animals and was followed by a decline. The leukocyte counts were higher in the transcolonic group animals than in the laparoscopy group at 2 h and on day 1; however the difference was already present at baseline. A rise in the platelet count occurred on day 7 in all animals with normalization by day 14. There were no statistically significant differences among the 3 animal groups (Figure 3).

## 4. Discussion

NOTES is still an experimental surgical approach [3, 14]. Even though a variety of animal procedures have been shown to be feasible and several laparoscopically assisted procedures have been performed in humans with an acceptable postoperative course, the efficiency and safety of NOTES as well as its expected benefits, including reduced systemic inflammatory response, remain to be shown. Wide adoption of NOTES will depend largely on confirmation of decreased invasiveness compared with standard available techniques [3].

Reduced physiological impact has previously been shown to play a major role in adoption of a surgical technique. In the 1980s when a totally new technique of gall-bladder surgery, laparoscopic cholecystectomy, was introduced, medical professionals expressed little interest in the new approach. Over the years, numerous studies have shown that laparoscopic surgery leads to a reduced inflammatory response compared with open surgery [15, 16]. Consequently, the importance of this technique has increased significantly, with laparoscopy now representing the gold standard for many abdominal surgery indications.

Several organs can serve as a site of access for transluminal procedures. The decision of which organ to use depends largely on the type of procedure. A transgastric approach enables good access to organs in the lower abdomen and pelvis, whereas transcolonic and transvaginal approaches offer good access to organs in the upper abdomen such as the gallbladder. The colonic approach may also be better for procedures in the retroperitoneum or in the colon itself. However, there is fear of infection due to the extensive bacterial colonization of the colon. This concern is also mirrored by a low patient preference for the transcolonic approach [17]. Nevertheless, all NOTES procedures pose a risk of inadvertent damage to surrounding organs and vessels by blind transluminal penetration.

In our study, we compared the two main NOTES approaches to the peritoneal cavity in terms of their feasibility, safety, and mainly their impact on the inflammatory response. Performing transgastric and transcolonic peritoneoscopy was feasible in all our animals. Transgastric peritoneoscopy has previously been done in animals and humans [2, 18]. Our peritoneoscopy was simplified to only visualize major intra-abdominal organs. The reason for this decision was to guarantee that the NOTES procedures and the laparoscopic control would be of the same duration. Complex procedures, such as salpingectomy or cholecystectomy, take always longer when performed by a transluminal approach [19]. The extent of the inflammatory response has, in fact, been shown to correlate with the length of the procedure [20].

OTSC was chosen for closure in our study because it is becoming widely accepted as a closure technique in transluminal endoscopy. It has been used in many NOTES animal studies and also in numerous human cases [21–23]. We were able to close the access defect in all animals. Clip application was faster in the colon because its wall is thinner, making grasping the edges of the incision easier and disengagement from the grasper less likely. Pulling the grasped edges into the cap was thus easier. All operated animals survived well without complications. The air leak test, clinical course, and endoscopic appearance at the end of the study period suggested good patency of closure. This was confirmed by histopathological examination that showed transmural healing.

We observed no macroscopic signs of infection at necropsy; however, in about a third of the NOTES animals in each group, there were microscopic signs of inflammation with purulent exudates and positive Gram staining, demonstrating the presence of bacteria. This finding is actually not surprising. Microscopic signs of bacterial inflammation following NOTES closure have been reported before. Renteln et al. reported small, localized perigastric abscesses in stomach OTSC closures in 2 of 10 animals [23]. The incidence, however, was smaller than when endoclips were used (3 of 10 animals had signs of diffuse peritonitis). In a study by Martínek et al., inflammation was present in 7 of 10 pigs with stomach OTSC closures and in 7 of 10 pigs with rectal OTSC closures [22]. The clinical significance of these findings is not clear and remains to be determined.

The incidence of adhesions in the groups was not significantly different; however 2 adhesions in the transcolonic animals were large and tight, fixing the site of closure in the colon to a small bowel loop. Detailed examination revealed no signs of enterocolonic fistula at the time of necropsy. The formation of adhesive disease can be viewed as a response of the body to focal inflammation and infection [24]. Furthermore, spillage of gastric contents has been shown to induce adhesive disease [25]. Romagnuolo et al. showed that the rate of adhesions was comparable between transgastric NOTES and laparoscopy for repair of inadvertent colon injury [24]. We observed no adhesions in our laparoscopic group. Absence of adhesions thus cannot be confirmed as an expected benefit of transluminal endoscopy.

TNF-*α* is an acute-phase cytokine, produced mainly by macrophages, that stimulates systemic inflammation as a response to surgical stress [26]. McGee et al. were the first to investigate TNF-*α* in peritoneoscopy pigs and reported significantly reduced levels of TNF-*α* on days 7 and 14 in the NOTES animals [11]. In a similar study published shortly afterwards, levels of TNF-*α* were significantly higher in the NOTES group, and the elevation persisted for 7 days [13]. In another study, Suzuki et al. found that NOTES transvaginal cholecystectomy induced a smaller increase of TNF-*α* than the laparoscopic procedure [27]. In our study, we observed constant TNF-*α* levels in all groups. Similarly, others have seen no differences between NOTES and laparoscopy in TNF-*α* [28–31]. For unknown reasons, the serum TNF-*α* concentrations in the transcolonic group were significantly higher than in the 2 other groups. However, this difference existed before the procedure and persisted throughout the study period. Similarly, increased levels of leukocytes, which persisted for one day, were present in the transcolonic animals. It is possible that TNF-*α* and leukocyte elevations were attributable to the bowel preparation, because that was the only experimental difference between the transcolonic NOTES and laparoscopy animals prior to the procedure.

IL-1*β* and IL-6 have been used to demonstrate the reduced inflammatory response of laparoscopic versus open surgery [15, 32]. In our study, however, measurable levels of IL-1*β* and IL-6 were present only in 7.3% and 43.3% of samples, respectively. Nevertheless, this finding is consistent with those of others who reported undetectable IL-1*β* or IL-6 serum levels in most or even all samples [11, 30, 31]. Either the sensitivity of our assay was not low enough to detect small amounts of cytokines or we did not evaluate our samples at an optimal time. IL-6 was found to be elevated for only up to 12 h after NOTES surgery that lasted for as long as 136 min [33]. Furthermore, we did not measure the cytokine levels in peritoneal fluid. In a study by Sood et al., IL-1*β* was detectable only in early lavage samples in some animals [30].

CRP is an acute-stress protein that responds to surgical stress, including laparoscopy [34]. Many studies investigating the inflammatory response to NOTES did not include CRP in their evaluation [11–13, 28, 35]. In our study, we observed an elevation of CRP on day 1 that persisted until day 7 and then declined to baseline levels by day 14. This transient elevation occurred in all 3 groups of animals with no statistically significant difference even though the rise of CRP levels was gradual in the TG animals and continued until day 7. The finding of a transient elevation of CRP is consistent with other reports [19, 33, 34, 36]. Martínek et al. and Freeman et al., in studies of ovariectomies in dogs and pigs, also found an early increase in CRP on days 1 and 2, which was greater in the NOTES animals than in laparoscopic controls [19, 33]. In contrast, a recent study of Bergström et al. reported lower CRP levels on day 1 following NOTES uterine horn resection [36]. Others investigators, such as Sohn et al. and Vieira et al., did not find significant differences in CRP levels after rectosigmoid resection or cholecystectomy in pigs [29, 31]. In the two studies showing significant increases in CRP, the procedures in the NOTES groups were longer than those in the controls; thus the possibility that the operating time influenced CRP levels cannot be ruled out.

A mild and transient increase in leukocyte counts following surgical trauma, including NOTES, has previously been shown. Similarly, we observed a mild elevation of leukocyte count in the first days after the procedure followed by a decline across all animal groups. Absence of leukocytosis throughout the experiment is an indirect confirmation of the absence of significant inflammation in our animals.

Changes in platelet levels have previously been reported in NOTES studies. Two studies showed decreased levels on day 2 following NOTES procedures. In one, the decrease occurred only in the NOTES group, and in the other, it was also seen in the control group. In contrast, we observed an increase of platelets on day 7. The reasons for this phenomenon are unclear, but a possible explanation is that the elevation was caused by inflammation that was undetected. Thrombopoietin, the main stimulant of platelet production, is elevated in inflammatory states. However, the elevated levels of platelets were transient, returning to baseline levels by day 14, and were present in all animals regardless of the type of procedure. It was thus unlikely to have occurred in response to NOTES.

Several study limitations should be noted. Although the animals used represent the best available surgical model for NOTES procedures because of their close similarity to humans, the implications for human medicine are uncertain. Next, our sample size was rather small as we followed the 3R principle (i.e., replacement, reduction, and refinement) for ethical experimental use of animals. Since we showed no statistically significant differences between the groups, there is a possibility of type II error. Furthermore, levels of two tested cytokines were undetectable in a large proportion of our samples, probably due to a low-sensitivity assay and the fact that no systemic inflammatory response syndrome was induced by surgery or infection. The low inflammatory response in our animals may have been caused by the fact that we performed only a 15-minute peritoneoscopy rather than a regular surgical procedure such as cholecystectomy. Finally, the relevance and clinical consequences of changes in cytokine level are unclear. Although, due to its anatomy, physiology, genetics, and size similarities to humans, the pig is a suitable biological model, a definitive answer to the question of whether NOTES is less invasive than open surgery will come from clinical trials in humans.

In conclusion, our study shows that transgastric and transcolonic peritoneoscopy are technically feasible, safe procedures. The OTSC closure was also technically feasible in all animals. However, it resulted in microscopic signs of inflammation and infection in about a third of the animals. Transgastric, transcolonic, and laparoscopic peritoneoscopy resulted in similar changes in serum inflammatory markers. Transcolonic NOTES was not associated with an increased incidence of inflammatory complications or an increased inflammatory response compared to transgastric NOTES. However, our findings also do not support the assumption that NOTES is less invasive than laparoscopy.

## Figures and Tables

**Table 1:** OTSC closure, healing, and complications of NOTES procedures.

	Transgastric (*n* = 10)	Transcolonic (*n* = 10)
Mean time of closure (min)	8.3	5.8
Transmural healing (*n*)	10	10
Adhesions, minor (*n*)	4	2
Adhesions, major (*n*)	0	2
Fistula (*n*)	0	0
Macroscopic signs of infection (*n*)	0	0
Microscopic signs of infection (*n*)	4	3
Gram-positive staining (*n*)	4	3
